# Thymus and activation-regulated chemokine (TARC)/CCL17 and IgE are associated with elderly asthmatics

**DOI:** 10.1186/s12979-018-0118-7

**Published:** 2018-05-05

**Authors:** Kyung Mi Jo, Hyo Kyung Lim, Jae Woong Sull, Eugene Choi, Ji-Sook Lee, Mee Ae Cheong, Min Hwa Hong, Yoori Kim, In Sik Kim

**Affiliations:** 1 Department of Senior Healthcare, BK21 plus program, Graduate School, Eulji University, Daejeon, 34824 Korea; 2 Department of Biomedical Laboratory Science, School of Medicine, Eulji University, 77, Gyeryoung-ro 771 beon-gil, Jung-Gu, Daejeon, 34824 Republic of Korea; 3 Department of Biomedical Laboratory Science, College of Health Science, Eulji University, Seongnam, 13135 Korea; 4 Department of Respiratory Internal Medicine, College of Medicine, Konyang University, Daejeon, 35365 Korea; 5 Department of Clinical Laboratory Science, Wonkwang Health Science University, Iksan, 54538 Republic of Korea

**Keywords:** Asthma, Aging, TARC, Allergen-specific IgE

## Abstract

**Background:**

The pathogenesis of asthma, which is an allergic lung disease, is associated with a variety of allergens such as house dust mite, pollen, and mould, IgE containing serum IgE and allergen-specific-IgE, and inflammatory cytokines including thymus and activation-regulated chemokine (TARC)/CCL17. Because aging is an essential factor in the pathogenesis of asthma, we examined biomarkers related to asthmatic subjects depending on age.

**Results:**

Physiological indices such as FEV1(forced expiratory capacity in 1 s), FEV1 (% predicted), and FEV1/FVC(forced vital capacity) (%) in asthmatic subjects were lower than those in normal subjects. Total IgE, Der p1 specific IgE, and Der f1 specific IgE were elevated in serum of asthmatics relative to normal individuals. Regulated on activation, normal T cell expressed and secreted (RANTES)/CCL5 in serum and interleukin 6 (IL-6), interleukin 8 (IL-8), monocyte chemoattractant protein (MCP)-1/CCL2, RANTES, and macrophage inflammatory protein (MIP)-1α/CCL3 in bronchoalveolar lavage fluid (BALF) of asthmatic subjects were higher than in normal individuals. Upon classification of experimental groups depending on age, physiological indices and Der p1-specific IgE (class) were decreased in middle aged adult and elderly adult groups relative to the young adult group. TARC levels in serum were strongly elevated in the elderly adult group relative to the young adult and the middle aged adult groups. TARC in serum was related to total IgE in serum in the elderly adult group.

**Conclusions:**

Taken together, although TARC in serum and BALF is not different between normal and asthmatic individuals, TARC increases in serum of elderly asthmatic subjects. The level of TARC has a positive effect on the level of IgE in the elderly adult group. These findings may help us better understand the relationship of pathogenesis of allergic diseases and aging.

**Electronic supplementary material:**

The online version of this article (10.1186/s12979-018-0118-7) contains supplementary material, which is available to authorized users.

## Background

Asthma is an allergic disease in the respiratory tract that is characterized by lung inflammation and mucus secretion resulting in airway obstruction, as well as allergen-specific IgE [1, 2]. Asthma is caused by a variety of elements including environmental, genetic and immunological factors. House dust mites (HDMs), including *Dermatophagoides pteronissinus* (DP) and *Dermatophagoides farinae* (DF), may be sources of many specific allergen proteins including Der p 1 and Der f 1 [3, 4]. More than half of asthmatics are sensitized to HDM and have elevated levels of HDM-specific IgE in their serum. Cytokine secretion, which is one of the most important allergic inflammatory responses, is increased by HDM via Toll-like receptor (TLR) and proteinase-activated receptor (PAR) [5, 6]. Cytokines including interleukin 4 (IL-4), interleukin 5 (IL-5), IL-6, IL-8, interleukin 10 (IL-10), monocyte chemoattractant protein (MCP)-1/CCL2, and thymus and activation-regulated chemokine (TARC)/CCL17 trigger secondary inflammatory events, aggravating asthma pathogenesis.

Aging is an unavoidable and complicated process characterized by progressive loss of functional activity, repair, and recovery. Interaction of the proinflammatory state with aging, which is very important, occurs via the inflamm-aging process [7, 8]. Aging studies have suggested that potential biomarkers containing proinflammatory cytokines, hypoxic indicators, and redox state may be related to inflammation-associated aging. Asthma in elderly subjects shows higher mortality than in younger members of the population, including children and younger adults, and is underdiagnosed by age-related alterations such as dyspnea, immunosenescence and decreased skin test sensitivity [9]. Although asthma in elderly subjects is associated with immunological and non-immunologic mechanisms, the exact interaction of aging and asthma has yet to be unveiled. In this study, we divided subjects into young adults, middle-aged adults, and elderly adults and studied the association of asthma and aging with biomarkers including cytokine and HDM-specific IgE in serum.

## Methods

### Study population

A total of 121 asthmatic subjects were randomly recruited from Konyang University Hospital according to the global initiative for asthma (GINA) guideline. The asthmatic subjects were classified as young adults (10–39 years), middle-aged adults (40–59 years), and elderly adults (≥ 60 years). Subjects were excluded if they had no history of smoking and other comorbidities. Additionally, 106 normal subjects were recruited as controls. The normal subjects had normal lung function, no history of asthma, and did not require medication.

### Collection of serum and BALF

Blood samples were collected and then centrifuged, after which the supernatant was separated from the samples. Following local anesthesia with lignocaine, sterile phosphate-buffered saline (PBS) (5 × 20 mL) was administered to lungs of normal (*n* = 9) and asthmatic (*n* = 39) individuals, after which the fluid was gently aspirated, pooled and collected into a tube. Nucleated cells in BALF and blood were counted using a Neubauer hemocytometer. Differential cell counts were performed from cytospin slides. Serum and BALF were stored at − 70 °C until used in this experiment.

### Laboratory investigations

White blood cell differential counts, hemoglobin and hematocrit levels were determined directly using a Sysmex XE-5000 system (Sysmex Corporation, Kobe, Japan). For measurement of serum HDM-specific IgE, serum was loaded into a Pharmacia Unicap 100 system (Pharmacia Unicap, Uppsala, Sweden). The DP or DF allergen covalently coupled to the cellulose solid-phase ImmunoCap and reacted with the specific IgE in the patient’s serum specimen. After washing, enzyme-labeled antibodies against IgE were added to form a complex, after which the IgE concentration was measured by fluorescence. HDM (DP or/and DF)-specific IgE+ is defined as > 0.35kU/L; class 0, < 0.35 kUA/L; class 1, 0.35 – < 0.7 kUA/L; class 2, 0.70 – < 3.5 kUA/L; class 3, 3.50 – < 17.5 kUA/L; class 4, 17.5 –< 50 kUA/L; class 5, 50 – ≤100 kUA/L; and class 6, > 100 kUA/L. Pulmonary function tests such as FEV1, FEV1 (% predicted), FVC, and FEV1/FVC were measured to determine the state of lung obstruction.

### Measurement of cytokine and chemokine concentrations

Concentrations of IL-4, IL-5, IL-6, IL-8, IL-10, MCP-1, tumor necrosis factor (TNF)-α, interferon (IFN)-γ, granulocyte macrophage colony simulation factor (GM-CSF), TARC, RANTES, and MIP-1α in serum were measured with a sandwich enzyme-linked immunosorbent assay (ELISA) using a cytokine measurement kit (BD Biosciences, San Diego, CA, USA; R&D Systems, Minneapolis, MN, USA) according to the manufacturer’s instructions. Briefly, 96-well plates were coated with 100 μL/well of appropriate monoclonal antibodies in 0.1 M carbonate buffer and incubated overnight at 4 °C, after which the plates were washed with PBS solution containing 0.05% Tween-20 and blocked with PBS solution with 5% bovine serum albumin (BSA) for 30 min at room temperature. Next, serum was added to the plates and incubated for 2 h at room temperature. The plates were then washed three times, after which they were incubated with appropriate secondary antibodies for 2 h at room temperature. The plates were then washed three times and incubated with substrate solution. Finally, the reaction was blocked by adding stop buffer and the absorbance was read at 450 nm.

### Statistical analysis

Data were presented as the means ± S.E.M. Statistical differences were analyzed using a Student’s t test for two-group comparisons. ANOVA was used to compare the three investigated age groups. Multiple comparisons were performed with Tukey’s post-hoc test. The Pearson correlation coefficient (*R*) was applied to present the strength of the relationship between variables. All analyses were conducted using the SPSS statistical software package (Version 20.0, Chicago, IL), with a *p* < 0.05 considered to be statistically significant.

## Results

### Characteristics of the study population

Physiology indices such as FEV1, FEV1 (% predicted) and FEV1/FVC in asthmatic subjects were decreased relative to normal subjects. Eosinophils in blood and BALF were increased in asthmatic subjects relative to normal subjects. Total IgE, Der p1-specific IgE and Der f1-specific IgE were increased in serum of asthmatic subjects relative to normal subjects (Table 1). Although Der p1-specific IgE (class) and Der f1-specific IgE (class) increased in the asthmatic group, the values did not differ significantly from those of the normal group. The level of MCP-1 and RANTES in serum and of IL-6, IL-8, MCP-1, RANTES and MIP-1α in BALF were significantly increased in the asthmatics relative to the normal subjects (Table 2).

**Table 1:** Characteristics of the study population

		Normal	Asthma
Number of subjects (female/male)		106(62/44)	121 (66/55)
Age (years)		45.3 ± 12.7(19~ 72)	50.8 ± 16.7 (15~ 84)
FEV1^§^		4.1 ± 0.9 (2.7~ 5.3)^**^	2.1 ± 0.8 (1~ 4)^**^
FEV1^§^ (% predicted)		101.6 ± 11.9 (87.1~ 121.3)^*^	81.4 ± 22.2 (34~ 125)^*^
FVC^∥^ (% predicted)		95.2 ± 11.5 (77.9~ 112.9)	91.3 ± 17.8 (40~ 140)
FEV1/FVC (%)		91.6 ± 4.9 (84.8~ 97.9)^**^	71.9 ± 14.2 (35~ 98)^**^
Blood cells	Neutrophils	55.3 ± 9.7 (28.5~ 75.4)	59.5 ± 14.3 (27~ 92)
	Lymphocytes	35.7 ± 9.3 (20~ 67.3)^**^	26.8 ± 11.0 (4~ 52)^**^
	Monocytes	6.7 ± 3.2 (2.0~ 19.0)^**^	7.0 ± 2.7 (1~ 17)^**^
	Eosinophils	2.0 ± 1.4 (0~ 6.0)^**^	5.7 ± 8.4 (0~ 55)^**^
	Basophils	0.3 ± 0.3(0~ 1.3)^*^	0.5 ± 0.5 (0~ 4)^*^
BALF cells	Neutrophils	2.0 ± 1.6 (0~ 5.1)^**^	16.6 ± 26.7 (0~ 90)^**^
	Lymphocytes	10.6 ± 8.4 (1.0~ 29.2)	16.9 ± 17.0 (0~ 69)
	Macrophage	86.2 ± 10.4 (62.0~ 99.0)^**^	59.9 ± 27.8 (3~ 95)^**^
	Eosinophils	0.2 ± 0.6 (0~ 1.9)	3.9 ± 8.5 (0~ 52)
	Basophils	0 ± 0 (0~ 0)	0.04 ± 0.1(0~ 1)
	Epithelial cells	0.8 ± 1.0 (0~ 2.5)	2.38 ± 9.6 (0~ 86)
	Total IgE	92.8 ± 143.4(2.2~ 694.2)^**^	376.4 ± 612.2 (2~ 3000)^**^
	Der p 1-specific IgE	0.8 ± 2.1(0~ 10.4)^*^	4.1 ± 15.3 (0~ 100)^*^
IgE	Der p 1-specific IgE (class)	0.4 ± 1.0(0~ 3)	0.62 ± 1.2 (0~ 5)
	Der f 1-specific IgE	1.5 ± 4.4(0~ 23.4)^**^	7.3 ± 19.5 (0~ 100)^**^
	Der f 1-specific IgE (class)	0.8 ± 1.2(0~ 4)	1.2 ± 1.5 (0~ 5)

FEV1^§^: forced expiratory volume in 1 second

FVC^∥^: forced vital capacity

Data are expressed as the means ± SD (the lowest value ~ the highest value)

*p < 0.05 and **p < 0.01 indicate statistically significant differences between the normal and asthma groups

**Table 2:** Measurement of cytokine concentration of the study population

		Normal	Asthma
Serum	IL-4	297.3 ± 1185.1 (0~ 7126.6)	342.0 ± 831.7 (0~ 4955)
	IL-5	136.2 ± 526.9 (0~ 2744.7)	189.7 ± 1184.7 (0~ 7399)
	IL-6	6.2 ± 22.2 (0~ 134.4)	11.0 ± 12.2 (0~ 50)
	IL-8	46.2 ± 234.8 (0~ 1460.9)	64.5 ± 155.5 (0~ 805)
	IL-10	29.9 ± 124.0 (0~ 723.2)	16.9 ± 77.6 (0~ 435)
	MCP-1	466.9 ± 860.4 (8.9~ 3032.4)^**^	2042.1 ± 1427.3 (246.9~ 8180.4)^**^
	TNF-α	87.2 ± 135.1 (8.5~ 884.3)^**^	9.5 ± 8.4 (0~ 38)^**^
	INF-γ	48.6 ± 161.8 (0~ 945.1)	52.6 ± 146.7(0~ 741)
	GM-CSF	31.1 ± 36.6 (13.1~ 185.1)	32.1 ± 5.1 (27.1~ 55.7)
	TARC	9.0 ± 23.9 (0~ 150.3)	4.9 ± 15.8 (0~ 64.7)
	RANTES	685.3 ± 56.6 (601.4~ 857.7)^**^	800.4 ± 98.0 (553.7~ 978.7)^**^
	MIP-1α	81.2 ± 91.5 (11.0~ 338.2)^**^	25.2 ± 43.7 (0~ 202.4)^**^
BALF	IL-4	26.8 ± 54.5 (0~ 144)	102.0 ± 191.2 (0~ 740)
	IL-5	0.0 ± 0.0 (0~ 0)	0.0 ± 0.0 (0~ 0)
	IL-6	1.7 ± 3.3 (0~ 8)^*^	11.2 ± 23.8 (0~ 89)^*^
	IL-8	470.9 ± 626.1 (0~ 2041)^**^	2682.1 ± 4417.5 (0~ 21,246)^**^
	IL-10	0.0 ± 0.0 (0~ 0)	0.0 ± 0.0 (0~ 0)
	MCP-1	197.7 ± 265.7 (0~ 701)^**^	936.7 ± 1268.5 (0~ 4452)^**^
	TNF-α	0.0 ± 0.0 (0~ 0)	0.0 ± 0.0 (0~ 0)
	INF-γ	9.0 ± 14.6 (0~ 39)	11.6 ± 15.6 (0~ 59)
	GM-CSF	0.0 ± 0.0 (0~ 0)	0.0 ± 0.0 (0~ 0)
	TARC	0.0 ± 0.0 (0~ 0)	2.7 ± 12.7 (0~ 73.1)
	RANTES	0.0 ± 0.0 (0~ 0)^**^	23.8 ± 40.1 (0~ 142.8)^**^
	MIP-1α	0.0 ± 0.0 (0~ 0)^**^	50.7 ± 84.9 (0~ 376.0)^**^

Moreover, MIP-1α in serum of the asthmatic group was lower than in normal. Cytokines did not differ in serum and BALF between atopic and non-atopic asthmatic groups (Additional file 1: Table S1).

### Different expression of measured parameters among normal and asthmatic age groups

The population of normal and asthmatic subjects was divided into three age groups, young adults (10–39 years), middle-aged adults (40–59 years) and elderly adults (60–83 years). Hb, Hct, ESR, Der p 1-specific IgE, and TARC were not significantly altered among three groups of normal (Additional file 1: Table S2). In asthmatic group, Hb and Hct were decreased in the elderly adult group, but ESR was increased in the elderly adult group relative to the young adult group. Indices such as FVC, FEV1 and FEV1/FVC were decreased in the elderly adult group relative to the young adult and middle-aged adult groups. The level of Der p1 IgE(class) in the middle-aged adult and elderly adult groups decreased significantly when compared to the young adult group. The level of serum TARC was markedly elevated in the elderly adult group relative to the young adult and the middle-aged adult groups (Table 3). The difference of TARC in each group between normal and asthmatic subjects is not significant (Additional file 1: Table S2).

**Table 3:** Different expression of the measured parameters among the asthmatic age groups

	Young adult group (*N* = 33,Ave 29.4 ± 6.7)	Middle-aged adult group (*N* = 49,Ave 50.1 ± 5.5)	Elderly adult group (N = 39,Ave 69.8 ± 5.9).	*P* value (Tukey HSD)
Hb	13.9 ± 2.0^$$^	13.6 ± 1.3	12.8 ± 1.3^$$^	0.007
Hct	41.2 ± 5.7^$^	40.1 ± 3.9	38.1 ± 4.0^$^	0.012
ESR	17.8 ± 17.3^$^	23.2 ± 24.3	34.7 ± 28.3^$^	0.036
Der p1-specific IgE(class)	1.12 ± 1.4*^$^	0.4 ± 1.1*	0.43 ± 1.1^$^	0.042, 0.050
FVC	3.6 ± 1.0^**$$^	2.9 ± 0.7^**^	2.4 ± 0.7^$$^	^$^0.000, ^*^0.001
FEV1	3.0 ± 0.7^**$$^	2.1 ± 0.6^**##^	1.5 ± 0.5^$$##^	^$^0.000, ^*^0.000, ^#^0.001
FEV1/FVC	80.0 ± 9.6^$$^	73.2 ± 13.1^##^	63.6 ± 14.8^$$##^	^$^0.000, ^#^0.004
Serum TARC	0.0 ± 0.0	0.0 ± 0.0^#^	12.9 ± 23.9^#^	^#^0.045

Data are expressed as the means ± SD

*p < 0.05 and **p < 0.01 indicate between the young adult group and middle-aged group

^#^*p* < 0.05 and ^##^*p* < 0.01 indicate between the middle aged group and elderly group

^$^*p* < 0.05 and ^$$^*p* < 0.01 indicate between the young adult group and elderly group

### Correlation among measured parameters in the total group and the elderly adult group of asthmatic subjects

The correlations among measured parameters in the total and the elderly adult groups are shown in Table 4. The serum TARC showed a positive correlation with total IgE in asthmatic subjects and the elderly adult group based on Pearson’s correlation coefficients. (0.3 < *r* < 0.7). Serum IL-6 showed a negative correlation with Der p1 specific IgE (class) in the total asthmatic group based on Pearson’s correlation coefficients (− 0.7 < *r* < − 0.3). However, serum IL-6 was not significantly correlated with Der p1 IgE(class) in the elderly adult group.

**Table 4:** Correlation among the measured parameters in the total group and the elderly adult group of asthmatic subjects

Total group	pb	Hct	ESR	FEV1/ FVC	Serum IL-6	Serum TARC
Total IgE	*R* = 0.282 *p* = 0.004^**^	*R* = 0.294 *p* = 0.004^**^	*R* = − 0.232 *p* = 0.050^*^	*R* = 0.049 *p* = 0.653	*R* = − 0.297 *p* = 0.111	*R* = 0.417 *p* = 0.022^*^
Der p1-specific gE	*R* = − 0.082 *p* = 0.388	*R* = − 0.099 *p* = 0.298	*R* = 0.047 *p* = 0.667	*R* = − 0.036 *p* = 0.713	*R* = − 0.223 *p* = 0.173	*R* = − 0.098 *p* = 0.553
Der p1-specific IgE(class)	*R* = − 0.032 *p* = 0.734	*R* = − 0.049 *p* = 0.611	*R =* − 0.009 *p* = 0.932	*R* = 0.014 *p* = 0.884	*R* = − 0.344 *p* = 0.032^*^	*R* = − 0.106 *p* = 0.519
Der f1-specific IgE	*R* = − 0.041 *p* = 0.670	*R* = − 0.058 *p* = 0.546	*R* = 0.013 *p* = 0.905	*R* = − 0.048 *p* = 0.631	*R* = − 0.218 *p* = 0.182	*R* = − 0.099 *p* = 0.550
Der f1-specific IgE(class)	*R* = 0.033 *p* = 0.733	*R* = 0.030 *p* = 0.755	*R* = − 0.114 *p* = 0.295	*R* = − 0.015 *p* = 0.876	*R* = − 0.160 *p* = 0.332	*R* = − 0.042 *p* = 0.799
Elderly adult group	Hb	Hct	ESR	FEV1/ FVC	S-IL-6	S-TARC
Total IgE	*R* = 0.219 *p* = 0.229	*R* = 0.176 *p* = 0.336	*R* = − 0.370 *p* = 0.069	*R* = 0.223 *p* = 0.246	*R* = − 0.442 *p* = 0.173	*R* = 0.630 *p* = 0.038^*^
Der p1-specific IgE	*R* = − 0.090 *p* = 0.612	*R* = − 0.119 *p* = 0.501	*R* = 0.066 *p* = 0.743	*R* = 0.143 *p* = 0.443	*R* = − 0.073 *p* = 0.797	*R* = − 0.006 *p* = 0.982
Der p1-specific IgE(class)	*R* = 0.128 *p* = 0.472	*R* = 0.094 *p* = 0.597	*R* = 0.049 *p* = 0.808	*R* = 0.185 *p* = 0.318	*R* = − 0.304 *p* = 0.271	*R* = 0.186 *p* = 0.507
Der f1-specific IgE	*R* = 0.050 *p* = 0.780	*R* = 0.034 *p* = 0.849	*R* = 0.021 *p* = 0.917	*R* = 0.272 *p* = 0.139	*R* = 0.034 *p* = 0.906	*R* = − 0.052 *p* = 0.853
Der f1-specific IgE(class)	*R* = 0.083 *p* = 0.643	*R* = 0.076 *p* = 0.668	*R* = − 0.280 *p* = 0.157	*R* = 0.377 *p* = 0.037^*^	*R* = − 0.287 *p* = 0.300	*R* = 0.086 *p* = 0.760

^*^correlation with *p* < 0.05

^**^correlation with *p* < 0.01

## Discussion

Asthma, which is one of the most prevalent allergic diseases, is caused by a variety of pathophysiological mechanisms [10]. HDMs are a major source of allergic asthma and DP and DF are of the most common HDMs [11]. IgE is considered a clear sign of allergic disease including asthma [11]. Der p 1 and Der f 1, major allergens proteins of HDMs, have a high IgE reactivity [12], and total IgE and Der p 1- or Der f 1–specific IgE are elevated in asthma [13]. In our study, total IgE, Der p1-specific IgE, and Der f1-specific IgE were elevated in asthmatic subjects when compared to normal subjects (Table 1). In a previous study, asthmatic subjects showed significantly more eosinophils, neutrophils, and cytokines such as IL-5, IL-6, IL-8, granulocyte colony stimulation factor (G-CSF) and RANTES in BALF when compared to healthy control subjects [14, 15]. As shown in Tables 1 and 2, neutrophils, IL-6, IL-8, RANTES, MCP-1, and MIP-1α in BALF of asthmatic subjects were also elevated relative to normal subjects. Both IL-8 and RANTES are major factors in increasing migration and proliferation of neutrophils and bronchial smooth muscle cells. These cytokines may be involved in increases in immune cells, particularly neutrophils. Although we also measured IL-5 and GM-CSF in BALF and serum. The difference between normal and asthmatic subjects was not significant.

Aging is an unavoidable and complicated process characterized by progressive loss of functional activity, repair and recovery [16]. As shown in Table 2, hematological indices such as Hb and Hct in the elderly adult group decreased significantly when compared to the young adult group, and physiological indices such as FVC, FEV1 and FEV_1_/FVC ratio in the elderly adult group showed stronger decreases than in the young adult group or middle-aged adult group. Aging is related to a progressive decline of lung function [17], and subjects with severe persistent asthma were much older than those with mild asthma [18]. Neutrophils were increased in the sputum of elderly asthma subjects. Therefore, neutrophilic airway inflammation is more common in elderly and severe asthma subjects [19, 20]. In our study, neutrophils in serum and BALF of asthmatic subjects were elevated when compared to normal subjects (Table 1). In the elderly adult group, neutrophils in serum and BALF were elevated as compared to the young adult and middle-aged adult groups, although this difference was not significant (data not shown). Accordingly, the relationship between neutrophils and aging after sub-classification should be analyzed further according to severity.

It is becoming recognized that the immune system declines with age via a process known as immunosenescence, which leads to a higher incidence of infections, neoplasia and autoimmune diseases [21]. The age-dependent decline in the immune system could be attributed to the functional activity of hematopoietic stem cells in older subjects [22]. Previous studies have reported that total serum IgE in the elderly adult group is lower than in the younger group and total IgE and allergen specific IgE have decreased with age in allergic patients as well as healthy members of the population [23]. In the present study, the level of total IgE in the elderly adult group was decreased relative to the young adult and middle-aged adult groups. However, this difference did not achieve statistical significance (data not shown). As shown in Table 3, Der p 1-specific IgE(class) was decreased in the middle-aged adult and the elderly adult groups relative to the young adult group. More than 31 allergens in the HDMs extracts have been reported to date, and specific IgE reactivity profiles to purified allergens vary in subjects from different countries [24]. Future investigations will need to be conducted to examine the association of other specific-IgEs with aging.

Although we could not detect statistically significant differences in serum TARC between normal subjects and asthmatic subjects, serum TARC was elevated in the elderly adult group relative to the young adult and the middle-aged adult groups, in which serum TARC was not detected (Table 3). Moreover, TARC was positively related to total IgE (Table 4). TARC plays a dominant role in Th_2_-type disease conditions by recruiting Th_2_ cells into inflammatory sites [25]. Several studies have demonstrated that there were elevated levels of TARC in patients with atopic dermatitis [26, 27] and asthma [28, 29]. The elevated TARC in asthma patients may be a reflection of increased TARC expression at inflammatory sites of the asthmatic airway [30]. The normal levels of serum TARC in healthy children and adults differ depending on age, with TARC levels being higher in children [31, 32].

However, in our study, the level only increased in the elderly adult group of the asthmatic subjects. It should be noted that this study had a few limitations. First, the asthmatic subjects included in this study show various allergic status (GINA 1–4). The relationship between severity and other parameters cannot be evaluated in this study. Second, we do not have any information regarding whether asthma found in elderly adult groups developed as a result of late onset asthma or was diagnosed in their childhood. Further study will be need to investigate these limitations.

## Conclusion

TARC in serum of the elderly adult group was increased when compared to members of other younger groups, and was positively related to total IgE in the total and elderly adult asthma group. Cytokines such as IL-6, IL-8, MCP-1, RANTES, and MIP-1α were significantly altered in the elderly adult group. The results presented herein will help elucidate the pathogenesis of allergic diseases such as asthma, as well as to mining of biomarkers associated with age.

## Additional file

Additional file 1: **Table S1.** Cytokine concentration in the atopic and non-atopic groups. **Table S2.** Different expression of the measured parameters among the normal age groups (DOCX 16 kb)

## Abbreviations

BALF: Bronchoalveolar lavage fluid
BSA: Bovine serum albumin
DF: *Dermatophagoides farina*
DP: *Dermatophagoides pteronissinus*
ELISA: Enzyme linked immunosorbent assay
FEV1: Forced expiratory capacity in 1 s
FVC: Forced vital capacity
HDMs: House dust mites
MCP: Monocyte chemoattractant protein
PAR: Proteinase activated receptor
PBS: Phosphate buffered saline
TARC: Thymus and activation regulated chemokine
TLR: Toll like receptor
