# Supplementary material for: Thymus and activation-regulated chemokine (TARC)/CCL17 and IgE are associated with elderly asthmatics
Source: Immun Ageing. 2018 May 5;15:13. doi: 10.1186/s12979-018-0118-7 (PMC5936629; doi:10.1186/s12979-018-0118-7)
Supplement: Supplementary file 1 — Table S1. Cytokine concentration in the atopic and non-atopic groups. Table S2. Different expression of the measured parameters among the normal age groups (DOCX 16 kb) [file 12979_2018_118_MOESM1_ESM.docx]

**Table S1. Cytokine concentration in the atopic and non-atopic groups**

|  | | Atopic group | Non Atopic group |
| --- | --- | --- | --- |
| Serume | IL-4 | 381.2±1131.5(0~4955) | 209.7±316.3(0~778) |
|  | IL-5 | 0.0±0.0(0~0) | 0.0±0.0(0~0) |
|  | IL-6 | 11.3±12.3(0~50) | 12.9±13.6(0~45) |
|  | IL-8 | 54.7±121.9(0~512) | 45.0±85.4(0~285) |
|  | IL-10 | 34.7±109.9(0~435) | 0.0±0.0(0~0) |
|  | MCP-1 | 2033.4±1679.7(246.9~8180.4) | 2397.3±1454.2(1057.5~6378.9) |
|  | TNF-α | 9.8±7.5(0~30) | 11.5±10.8(0~38) |
|  | INF-γ | 61.8±169.1(0~741) | 21.8±18.5(0~55) |
|  | GM-CSF | 32.6±6.7(27.1~55.7) | 31.1±3.6(28.4~41.1) |
|  | TARC | 6.9±20.2(0~64.7) | 0.0±0.0(0~0) |
|  | RANTES | 788.8±74.7(609.7~923.7) | 803.0±123.6(553.7~978.7) |
|  | MIP-1α | 27.7±47.5(0~202.4) | 27.5±49.1(0~163.8) |
| BALF | IL-4 | 92.3±169.9(0~740) | 103.9±218.6(0~734) |
|  | IL-5 | 0.0±0.0(0~0) | 0.0±0.0(0~0) |
|  | IL-6 | 18.2±31.0(0~89) | 6.3±14.2(0~39) |
|  | IL-8 | 3856.5±5603.7(0~21246) | 1761.7±3064.1(0~10327) |
|  | IL-10 | 0.0±0.0(0~0) | 0.0±0.0(0~0) |
|  | MCP-1 | 1208.7±1297.1(0~4452) | 743.1±1421.9(0~4384) |
|  | TNF-α | 2.1±9.3(0~41) | 1.2±4.0(0~13) |
|  | INF-γ | 13.1±16.7(0~59) | 10.2±16.2(0~44) |
|  | GM-CSF | 0.0±0.0(0~0) | 0.0±0.0(0~0) |
|  | TARC | 1.7±7.7(0~33.9) | 6.6±22.0(0~73.1) |
|  | RANTES | 24.9±38.2(0~142.8) | 16.5±34.7(0~1138) |
|  | MIP-1α | 35.1±47.8(0~167.3) | 68.5±126.2(0~376.0) |

**Table S2. Different expression of the measured parameters among the normal age groups**

|  | Young adult group  (N=55,Ave 24.1±4.3) | Middle-aged adult group (N=31,Ave 50.9±5.5) | Elderly adult group  (N=20,Ave 64.9±4.2). | *P* value  (Tukey HSD) |
| --- | --- | --- | --- | --- |
| Hb | 14.0±1.1 | 14.7±1.4 | 13.7±1.5 |  |
| Hct | 41.8±2.0 | 43.8±2.9 | 41.9±4.5 |  |
| ESR | 2.6±2.6 | 3.4±1.7 | 7.0±4.5 |  |
| Der p1-specific IgE(class) | 0.54±1.0 | 0.0±0.0 | 0.47±1.1 |  |
| Serum TARC | 28.8±57.2 | 2.8±11.1 | 3.4±2.9 |  |
